# Barriers to Maternal Presence at the Bedside of Infants With Neonatal Opioid Withdrawal Syndrome

**DOI:** 10.7759/cureus.117298

**Published:** 2026-10-01

**Authors:** Amanda Guth, Angelica Gangemi-Hanninen, Himani Divatia

**Affiliations:** 1 Internal Medicine - Pediatrics, Christiana Care Health System, Newark, USA

**Keywords:** eat-sleep-console, maternal-child health, neonatal opioid withdrawal syndrome, public health interventions, qualitative interviews

## Abstract

Background

Neonatal opioid withdrawal syndrome (NOWS) occurs in infants exposed to opioids during pregnancy. Caregiver presence is an important component of family-centered NOWS care. This study compared documented caregiver bedside presence and clinical outcomes before and after implementation of the Eat, Sleep, Console (ESC) approach and explored barriers to bedside presence reported by mothers in the ESC cohort.

Methods

We compared a retrospective non-ESC cohort of infants with NOWS admitted to an academic general pediatrics floor from August through December 2021 with a prospective ESC cohort admitted from May through August 2022. Clinical variables included length of stay (LOS), morphine receipt and duration, adjuvant pharmacologic therapy, and documented caregiver bedside presence. Mothers in the ESC cohort were invited to provide exploratory feedback regarding barriers to bedside presence.

Results

Fifty-four infants were included (41 non-ESC; 13 ESC). No statistically significant differences were observed in LOS, morphine receipt, morphine duration, adjuvant pharmacologic therapy, or documented caregiver bedside presence. Four of 13 mothers in the ESC cohort completed follow-up; two described concerns related to communication, shared decision-making, and perceived stigma.

Conclusion

Clinical outcomes and documented caregiver bedside presence did not differ significantly between cohorts. Maternal feedback was limited and should be considered exploratory, but it identified provider-family communication and support as potential areas for further study. Larger prospective studies with more precise measurement of caregiver presence and systematic assessment of family experiences are needed.

## Introduction

Neonatal opioid withdrawal syndrome (NOWS) occurs in infants born to mothers who used opioids during pregnancy [1]. NOWS presents as a constellation of neurologic, gastrointestinal, and musculoskeletal symptoms and can have long-lasting effects on infants' growth and development beyond the acute withdrawal period. Maternal prenatal polysubstance use and continuous exposure to high-dose opioids in utero contribute to unfavorable neonatal outcomes among infants diagnosed with NOWS [2,3]. In addition to prenatal exposures, social factors play an important role in determining NOWS severity. Notably, limited parental presence at an infant's bedside during hospitalization has been associated with poorer outcomes among infants with NOWS [4]. Infants whose caregivers were less present during hospitalization have been shown to experience longer hospital stays and prolonged pharmacologic treatment, indicating more severe withdrawal symptoms [4].

Historically, management of NOWS was guided by the Finnegan Neonatal Abstinence Scoring System (FNASS), which requires periodic assessments of clinical signs and symptoms by members of the clinical team [5]. Because FNASS relies on subjective scoring, variability in assessment may contribute to inconsistencies in treatment decisions and pharmacologic management. When withdrawal symptoms are not adequately controlled with first-line opioid therapy (commonly morphine or methadone), additional medications such as clonidine or phenobarbital may be used as adjuvant pharmacologic therapy to further manage withdrawal symptoms [6]. In response to these limitations and the desire to reduce pharmacologic exposure, the Eat, Sleep, Console (ESC) approach was introduced as a caregiver-centered model of NOWS management that prioritizes non-pharmacologic care and functional assessment of the infant. The ESC protocol emphasizes three core components: 1) Eat: parents are encouraged to feed infants on demand with breast milk or formula, ensuring adequate nutrition and minimizing irritability related to hunger; 2) Sleep: caregivers help create a calm, soothing environment that promotes regular and restorative sleep, which is often disrupted during withdrawal; and 3) Console: parents are taught techniques such as swaddling, gentle rocking, and the use of pacifiers to comfort infants experiencing withdrawal symptoms.

Through this caregiver-centered approach, parents are taught how to recognize and respond to their infant's needs while minimizing the use of pharmacologic therapy [7]. Studies evaluating the ESC model have demonstrated reductions in medication use, length of hospital stay, and time to medical readiness for discharge [8]. Importantly, the success of the ESC model relies heavily on consistent caregiver presence at the bedside [9].

Despite the benefits of caregiver-centered care, several barriers can limit parental presence during an infant's hospitalization. Transportation challenges, additional childcare responsibilities, and the need for off-site methadone dosing can disrupt parental involvement. Additionally, the requirements of residential substance use disorder treatment programs, combined with the stigma and guilt experienced by parents with substance use disorders while witnessing their infants undergo withdrawal, further complicate sustained bedside presence [4]. While previous studies have identified some of these barriers, further research is needed to better understand and address the systemic challenges these parents face.

Recognizing the importance of caregiver involvement in the ESC approach and the barriers that may limit bedside presence during hospitalization, this study had two objectives. First, we compared documented caregiver bedside presence and clinical outcomes between infants managed before implementation of ESC and infants managed using the ESC protocol. Second, as an exploratory qualitative objective, we sought to describe barriers to bedside presence reported by mothers in the ESC cohort and identify potential opportunities to support family involvement at our institution. Throughout the manuscript, "caregiver" refers broadly to individuals documented as present at the infant's bedside, whereas "mother" or "maternal" refers specifically to participants in the qualitative questionnaire component.

## Materials and methods

This single-center cohort study included infants diagnosed with NOWS who were admitted to the ChristianaCare General Pediatrics Floor. All eligible infants admitted during the predefined study periods were included. The retrospective, non-ESC cohort consisted of infants admitted between August and December 2021, prior to implementation of the ESC protocol. The prospective ESC cohort included infants admitted between May and August 2022 following implementation of ESC. Infants were eligible for inclusion if they were diagnosed with NOWS and admitted to the general pediatrics floor during the respective study period. Infants requiring neonatal intensive care unit (NICU) admission or placed in Division of Family Services (DFS) custody during hospitalization were excluded.

Medical record review was performed for both cohorts. Data collected included infant demographic and clinical characteristics, including sex, gestational age, and hospital length of stay; maternal substance use and use of medication-assisted treatment (MAT); infant receipt and duration of morphine therapy; use of adjuvant pharmacologic therapy; and caregiver presence at the infant's bedside. Caregiver presence was analyzed as a binary variable indicating whether bedside presence was documented during the hospitalization; the available data did not permit reliable quantification of the total duration or proportion of hospitalization spent at the bedside. In the prospective cohort, caregivers were additionally asked to document bedside presence using a sign-in sheet. Infants in the prospective cohort were managed using the ESC protocol, which emphasizes nonpharmacologic care, functional assessment, and caregiver involvement in the management of withdrawal symptoms. Parents received education and guidance from the healthcare team regarding feeding, recognition of infant cues, soothing and consoling techniques, environmental modification, and other supportive strategies. When caregivers were unable to remain at the bedside, nursing staff continued ESC-based supportive measures to provide consistency in care.

Mothers in the prospective ESC cohort were invited to complete a questionnaire at discharge or were contacted by telephone following discharge to identify barriers that limited their ability to remain at the bedside and to obtain qualitative feedback regarding interventions that could have facilitated greater caregiver presence (Appendix 1). Of the 13 mothers in the ESC cohort, four were successfully contacted and provided follow-up feedback. Questionnaire responses were reviewed descriptively to identify reported barriers and recurring concerns; given the small number of respondents, no formal qualitative thematic analysis was performed. The questionnaire was developed by the study authors based on a review of the literature and discussions with mothers of infants with NOWS regarding barriers to bedside presence prior to study implementation. It was subsequently reviewed and refined by the study investigators and vetted by a multidisciplinary team of physicians, nurses, and social workers on the general pediatrics floor to assess the relevance, clarity, and appropriateness of the questions prior to distribution. The questionnaire was developed specifically for this quality improvement initiative and did not undergo formal psychometric validation.

Potential confounding factors considered when interpreting differences between cohorts included gestational age, infant sex, maternal substance exposure, maternal MAT use, and caregiver presence, as these factors could independently influence NOWS severity, pharmacologic treatment requirements, and length of hospitalization. Given the limited sample size, adjusted multivariable analyses were not performed.

Continuous variables were summarized using descriptive statistics, including means and standard deviations and, where appropriate, medians and interquartile ranges. Hospital length of stay and duration of morphine therapy were compared between cohorts using the Mann-Whitney U test. Categorical variables were summarized as frequencies and percentages. Differences in receipt of morphine and adjuvant pharmacologic therapy were assessed using Fisher's exact test, while caregiver presence at the bedside was compared using the chi-square test. Statistical analyses were performed using the Statistical Analysis System (SAS). The final analytic sample included 54 infants, with 41 infants in the retrospective non-ESC cohort and 13 infants in the prospective ESC cohort. Morphine-use data were available for 53 infants (41 non-ESC and 12 ESC), and morphine-duration data were available for 22 infants who received morphine (16 non-ESC and six ESC). No formal a priori sample size calculation was performed; rather, the sample consisted of all eligible infants meeting study criteria during the predefined study periods. The statistical output confirms the missingness underlying those morphine analyses and the Mann-Whitney comparisons.

This project was reviewed by the ChristianaCare Institutional Review Board Office and was determined to be a quality improvement initiative that did not meet the federal definition of research pursuant to 45 CFR 46.102(l); therefore, review and approval by the Institutional Review Board were not required.

## Results

A total of 54 infants met the inclusion criteria, including 41 infants in the retrospective non-ESC cohort and 13 infants in the prospective ESC cohort. Demographic and clinical characteristics of the two cohorts are summarized in Table 1. Mean gestational age was 38 weeks (range, 35-41) in the non-ESC cohort and 39 weeks (range, 36-40) in the ESC cohort. Mean hospital length of stay was 13.0 days in the non-ESC cohort and 11.8 days in the ESC cohort. There was no statistically significant difference in hospital length of stay between the two cohorts (Mann-Whitney U test, p = 0.31).

**Table 1:** Demographic and clinical characteristics of infants in the non-ESC and ESC cohorts Data are presented as n/N (%) for categorical variables and mean ± standard deviation (SD), mean (range), or median (interquartile range) for continuous variables, as indicated. Between-group comparisons of length of stay and morphine duration were performed using the Mann-Whitney U test. Fisher's exact test was used to compare morphine use and adjuvant pharmacologic therapy, and the chi-squared test was used to compare caregiver presence at the bedside. A two-sided p < 0.05 was considered statistically significant. MAT - medication-assisted treatment; ESC - East, Sleep, Console

Characteristic	Non-ESC cohort (N = 41)	ESC cohort (N = 13)	p-value
Sex, n (%)			-
Male	16 (39.0%)	8 (61.5%)	-
Female	25 (61.0%)	5 (38.5%)	-
Gestational age (weeks), mean (range)	38 (35–41)	39 (36–40)	-
Length of stay (days), mean ± SD	13.0 ± 9.3	11.8 ± 11.4	0.31
Morphine use, n/N (%)	16/41 (39.0%)	6/12 (50.0%)	0.52
Morphine duration (days), mean ± SD; median (IQR)	16.8 ± 6.2; 18.0 (9.0)	20.0 ± 9.9; 20.0 (18.0)	0.48
Mother using MAT, n (%)	28 (68.3%)	9 (69.2%)	-
Adjuvant pharmacologic therapy, n/N (%)	1/41 (2.4%)	2/13 (15.4%)	0.14
Caregiver present at bedside, n/N (%)	20/41 (48.8%)	6/13 (46.2%)	0.87

Morphine-use data were available for 53 of 54 infants (41/41 non-ESC and 12/13 ESC). For one infant in the ESC cohort, morphine-use data were missing from the extracted EHR dataset and could not be determined from the available data; therefore, analyses of morphine receipt were performed using the available-case denominator for each cohort. Morphine was required in 16 of 41 infants (39.0%) in the non-ESC cohort and six of 12 infants (50.0%) in the ESC cohort, with no statistically significant difference between groups (Fisher's exact test, p = 0.52). Morphine-duration data were available for 22 infants who received morphine (16 non-ESC and 6 ESC). The mean duration of morphine therapy was 16.8 days in the non-ESC cohort and 20.0 days in the ESC cohort. The median duration was 18.0 days in the non-ESC cohort and 20.0 days in the ESC cohort, with no statistically significant difference between groups (Mann-Whitney U test, p = 0.48).

Adjuvant pharmacologic therapy was administered to one of 41 infants (2.4%) in the non-ESC cohort and two of 13 infants (15.4%) in the ESC cohort. This difference was not statistically significant (Fisher's exact test, p = 0.14). Caregiver presence at the bedside was documented for 20 of 41 infants (48.8%) in the non-ESC cohort and 6 of 13 infants (46.2%) in the ESC cohort, with no statistically significant difference between groups (chi-square test, p = 0.87).

Among the 13 mothers in the prospective ESC cohort, four were successfully contacted for follow-up. Two of the four mothers reported limited bedside presence related to gaps in communication regarding delivery expectations and their infant's care plan, shared decision-making, and perceived stigma related to opioid use disorder. Qualitative feedback also highlighted the importance of regular communication between healthcare providers and families and the potential value of additional bedside education to support parental involvement.

## Discussion

In this single-center before-and-after cohort study, no statistically significant differences were observed between the non-ESC and ESC cohorts in hospital length of stay, morphine receipt, duration of morphine therapy, adjuvant pharmacologic therapy, or documented caregiver bedside presence. Documented caregiver presence was similar between cohorts (48.8% in the non-ESC cohort and 46.2% in the ESC cohort; p = 0.87). Adjuvant pharmacologic therapy was used in 2.4% of infants in the non-ESC cohort and 15.4% of infants in the ESC cohort; this difference was not statistically significant (p = 0.14). Given the small and unequal cohort sizes and unadjusted before-and-after design, these findings should be interpreted descriptively and do not establish equivalence between the cohorts or a causal effect of ESC implementation.

Caregiver involvement remains an important component of nonpharmacologic NOWS management. Previous research has associated greater caregiver presence with shorter hospitalization and decreased pharmacologic treatment [4]. In the present study, however, the available measure of caregiver presence was binary and did not quantify the amount of time caregivers spent at the bedside. Accordingly, the similar proportions of documented caregiver presence between cohorts should not be interpreted as evidence that ESC increased or decreased caregiver involvement. More granular prospective measurement of caregiver presence will be necessary to evaluate this relationship.

The exploratory maternal questionnaire provided additional context regarding potential barriers to bedside presence. Only four of 13 mothers in the ESC cohort completed follow-up, and two described concerns related to communication about delivery expectations and the infant's care plan, shared decision-making, and perceived stigma. These individual experiences identify potential areas for further investigation but cannot establish the prevalence of these barriers, demonstrate provider bias, or determine their effect on caregiver presence or infant outcomes. Nonetheless, they are consistent with literature emphasizing the importance of social and emotional support for families affected by NOWS [4]. Family-centered strategies such as clear expectations regarding care plans, opportunities for questions, regular check-ins, and methods to confirm understanding may warrant further evaluation in this population.

The findings should also be considered in the context of the broader healthcare environment. Prior literature has described stigma toward individuals with substance use disorders and its potential effects on healthcare experiences [10]. Our study was not designed to directly evaluate provider behavior or unconscious bias, and the limited questionnaire responses do not permit conclusions regarding whether bias occurred in this cohort. Future studies could more directly examine provider-family communication, perceived stigma, and their relationships with caregiver engagement using validated instruments and larger prospective samples.

Several limitations must be acknowledged. First, this was a single-center quality-improvement study with small and unequal cohort sizes (41 non-ESC and 13 ESC infants), limiting statistical power and generalizability. The nonrandomized before-and-after design used historical rather than concurrent comparison groups, creating the possibility that temporal changes in clinical practice or other unmeasured factors contributed to observed differences. Although potential confounders including gestational age, infant sex, maternal substance exposure, maternal MAT use, and caregiver presence were considered, the limited sample size precluded multivariable adjustment; causal effects of ESC therefore cannot be inferred. Additionally, morphine-use data were missing for one infant in the ESC cohort in the extracted EHR dataset and could not be determined from the available data; therefore, the morphine-use analysis was based on available cases. Second, caregiver bedside presence was recorded as a binary documented measure, and the available data did not reliably quantify duration or proportion of hospitalization spent at the bedside. Interdisciplinary documentation and data collection may also have affected the completeness of this measure. Third, only four of 13 mothers in the ESC cohort completed follow-up, creating substantial potential for nonresponse and selection bias; mothers who were reached may not represent the experiences of the full cohort. Exclusion of infants placed in DFS custody may further limit the range of family circumstances represented. The interval between hospitalization and some follow-up contacts may also have introduced recall bias. Finally, the questionnaire was developed for this quality-improvement initiative and did not undergo formal psychometric validation. Larger prospective studies using concurrent comparison groups, standardized measures of caregiver presence, validated family-experience instruments, and adjusted analyses are needed to more rigorously evaluate caregiver involvement, family experiences, and clinical outcomes associated with NOWS care.

## Conclusions

In this exploratory single-center quality-improvement study, no statistically significant differences were observed between the non-ESC and ESC cohorts in hospital length of stay, morphine use, morphine duration, adjuvant pharmacologic therapy, or documented caregiver bedside presence. The exploratory questionnaire responses from four mothers identified communication, shared decision-making, and perceived stigma as potential areas for further investigation; however, the limited response rate precludes conclusions regarding the prevalence of these experiences or their effects on caregiver presence and clinical outcomes. These findings highlight the need for larger prospective studies with more precise measurement of caregiver presence and systematic assessment of family experiences to evaluate strategies that support family-centered care for infants with NOWS.

## Figures and Tables

**Figure 1:**
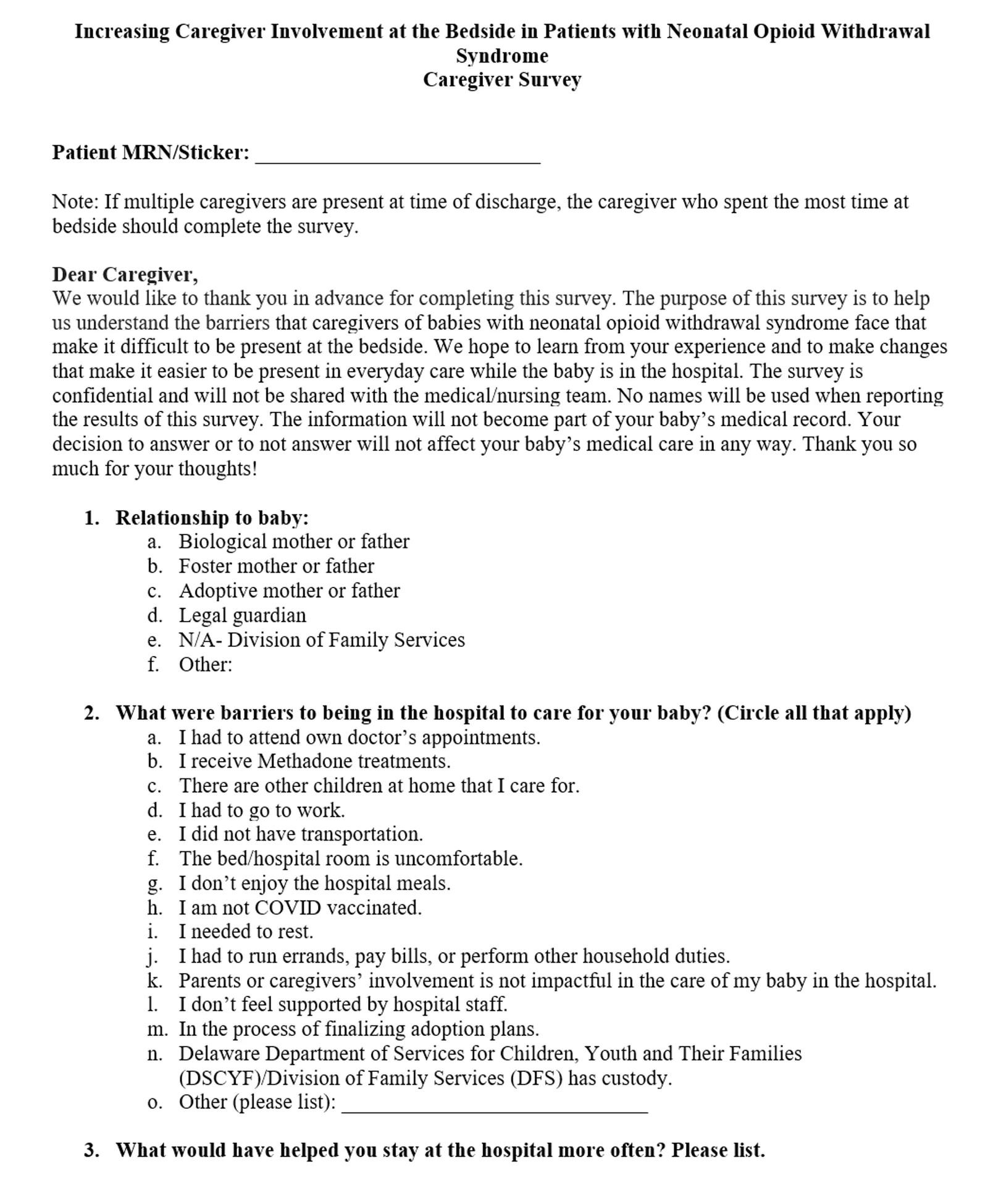
Increasing caregiver involvement at the bedside in patients with neonatal opioid withdrawal syndrome caregiver survey The questionnaire was developed by the study authors specifically for this quality improvement initiative based on relevant literature and discussions with mothers of infants with neonatal opioid withdrawal syndrome (NOWS) regarding barriers to bedside presence. The questionnaire was reviewed by the study investigators and a multidisciplinary team of physicians, nurses, and social workers on the general pediatrics floor prior to distribution. The questionnaire was not adapted from an external instrument and did not undergo formal psychometric validation.
